# Multiparameter analysis of homogeneously R-CHOP-treated diffuse large B cell lymphomas identifies CD5 and FOXP1 as relevant prognostic biomarkers: report of the prospective SAKK 38/07 study

**DOI:** 10.1186/s13045-015-0168-7

**Published:** 2015-06-14

**Authors:** Alexandar Tzankov, Nora Leu, Simone Muenst, Darius Juskevicius, Dirk Klingbiel, Christoph Mamot, Stephan Dirnhofer

**Affiliations:** Institute of Pathology, University Hospital Basel, Schoenbeinstrasse 40, CH-4031 Basel, Switzerland; Swiss Group for Clinical Cancer Research (SAKK), Effingerstrasse 40, CD-3008 Bern, Switzerland; Division of Hematology and Oncology, Cantonal Hospital Aarau, Tellstrasse house Nr. 40, CH-5001 Aarau, Switzerland

**Keywords:** DLBCL, Prognosis, Phenotype, FISH, Prospective trial, CD5, FOXP1

## Abstract

**Background:**

The prognostic role of tumor-related parameters in diffuse large B cell lymphoma (DLBCL) is a matter of controversy.

**Methods:**

We investigated the prognostic value of phenotypic and genotypic profiles in DLBCL in clinical trial (NCT00544219) patients homogenously treated with six cycles of rituximab, cyclophosphamide, hydroxydaunorubicin, vincristine, prednisone (R-CHOP), followed by two cycles of R (R-CHOP-14). The primary endpoint was event-free survival at 2 years (EFS). Secondary endpoints were progression-free (PFS) and overall survival (OS). Immunohistochemical (bcl2, bcl6, CD5, CD10, CD20, CD95, CD168, cyclin E, FOXP1, GCET, Ki-67, LMO2, MUM1p, pSTAT3) and in situ hybridization analyses (*BCL2* break apart probe, *C-MYC* break apart probe and *C-MYC/IGH* double-fusion probe, and Epstein–Barr virus probe) were performed and correlated with the endpoints.

**Results:**

One hundred twenty-three patients (median age 58 years) were evaluable. Immunohistochemical assessment succeeded in all cases. Fluorescence in situ hybridization was successful in 82 instances. According to the Tally algorithm, 81 cases (66 %) were classified as non-germinal center (GC) DLBCL, while 42 cases (34 %) were GC DLBCL. *BCL2* gene breaks were observed in 7/82 cases (9 %) and *C-MYC* breaks in 6/82 cases (8 %). “Double-hit” cases with *BCL2* and *C-MYC* rearrangements were not observed. Within the median follow-up of 53 months, there were 51 events, including 16 lethal events and 12 relapses. Factors able to predict worse EFS in univariable models were failure to achieve response according to international criteria, failure to achieve positron emission tomography response (*p* < 0.005), expression of CD5 (*p* = 0.02), and higher stage (*p* = 0.021). Factors predicting inferior PFS were failure to achieve response according to international criteria (*p* < 0.005), higher stage (*p* = 0.005), higher International Prognostic Index (IPI; *p* = 0.006), and presence of either *C-MYC* or *BCL2* gene rearrangements (*p* = 0.033). Factors predicting inferior OS were failure to achieve response according to international criteria and expression of FOXP1 (*p* < 0.005), cyclin E, CD5, bcl2, CD95, and pSTAT3 (*p* = 0.005, 0.007, 0.016, and 0.025, respectively). Multivariable analyses revealed that expression of CD5 (*p* = 0.044) and FOXP1 (*p* = 0.004) are independent prognostic factors for EFS and OS, respectively.

**Conclusion:**

Phenotypic studies with carefully selected biomarkers like CD5 and FOXP1 are able to prognosticate DLBCL course at diagnosis, independent of stage and IPI and independent of response to R-CHOP.

**Electronic supplementary material:**

The online version of this article (doi:10.1186/s13045-015-0168-7) contains supplementary material, which is available to authorized users.

## Background

Diffuse large B cell lymphoma (DLBCL) is the most common nodal lymphoid malignancy, comprising approximately 30 % of all adult lymphomas, with a rapidly rising incidence [1, 2]. DLBCL demonstrates an aggressive clinical course, but potentially 60–70 % of patients can be cured with the established rituximab, cyclophosphamide, hydroxydaunorubicin, vincristine, prednisone (R-CHOP) treatment standard [3]. Prediction of survival and stratification of patients for risk-adjusted therapy is based on the International Prognostic Index (IPI) [4]. R-CHOP has not only led to a marked improvement of survival in DLBCL but has also called into question the significance of the IPI [5], leading to introduction of the revised IPI (R-IPI) [6]. Recent data suggests that IPI and R-IPI no longer reliably identify DLBCL risk groups with a <50 % chance of survival, despite about 30–40 % of patients will still die of/with disease. Thus, there is a need for additional, particularly tumor-related, prognostic (and predictive) factors in DLBCL [7].

To date, only a limited number of tumor-related prognostic parameters exist for DLBCL like presence of *C-MYC* rearrangements or co-expression of bcl2 and c-myc. The morphological heterogeneity of DLBCL is reflected by significant molecular diversity at the genotypic, gene expression, and phenotypic levels [8, 9]. Gene expression profiling data convincingly showed that DLBCLs are derived from germinal center B cells (GCB) or activated B cells (ABC) [9–11]. Although the scientific evidence is robust and prognostically relevant, its translation into daily practice remains impractical because of the required high standard of tissue preservation, procedure duration, and costs. This problem prompted the search for molecular prognostic markers applicable to routine biopsies from patients with DLBCL. As a result, a large body of surrogate (phenotypic) models and algorithms to identify GCB and non-GCB DLBCL have been proposed and linked to outcomes [12]. Unfortunately, reliability and reproducibility of these models is often poor, impeding their translation into standard practice to predict survival and stratify patients for risk-adjusted therapy [12–14]. Technical issues, poor study designs, lack of standardization of evaluation procedures, and, particularly, lack of prospective trials all prevent an efficient clinical translation. A PubMed search for “DLBCL,” “R-CHOP,” “prognostic,” “marker,” and “prospective” identifies only a few prospective studies, in which biomarkers have been considered (e.g., [15–24]). Thus, there is an unmet requirement for further marker validation in prospective trials.

The translational study of the clinical trial “SAKK 38/07 Prospective evaluation of the prognostic value of positron emission tomography (PET) in patients with diffuse large B-cell-lymphoma under R-CHOP-14. A multicenter study” offered a unique opportunity to prospectively analyze the prognostic and predictive value of phenotypic and genotypic biomarkers suggested to play a prognostic role in DLBCL on a well-documented and homogenously treated clinical trial collective.

## Materials and methods

### Patient recruitment, selection, and treatment

The recruitment of patients for the SAKK 38/07 study started in November 2007 and finished in June 2010. Evaluation of the prognostic value of metabolic responses, as assessed by early PET after two cycles of R-CHOP-14, to identify a poor outcome patient subgroup was the main objective. PET was performed before, after two cycles of therapy, and at the end of treatment and was evaluated according to a 5-point scoring system with a cutoff determining positivity being set at 4 points (moderately increased uptake compared with the liver) [25]. The primary endpoint was event-free survival (EFS) at 2 years, and the secondary endpoints were progression-free (PFS) and overall survival (OS) after 2 and 5 years as well as the objective responses according to international criteria [26]. In accordance with the statistical advice for reaching sufficient power to address the two endpoints, recruitment of 154 patients was aimed. Because of concurrent registrations on the last recruitment day, 156 instead of 154 patients were recruited. Inclusion criteria were histologically proven diagnosis of CD20-positive DLBCL (no pretreatment revision of the slides by an expert hematopathologist was planned) including all Ann Arbor stages, tumor size >14 mm on CT or MRI (because lymph nodes ≥15 mm are considered “pathologic” on computerized imaging), PET positivity of the tumors (documented 2 weeks to 4 days prior to registration), performance status 0–2 on the ECOG scale, age >17, as well as no evidence of symptomatic central nervous system (CNS) disease, HIV, and/or hepatitis infection [27]. The study treatment consisted of R-CHOP given for six cycles followed by additional two applications of rituximab every 2 weeks (R-CHOP-14). Additionally, G-CSF support was given. The patients were asked to provide informed consent for the study and, separately, for the translational research. The primary pathology institutions were asked to send representative paraffin blocks for translational research after accomplishing the in-house diagnostic procedures to the Institute of Pathology at the University Hospital Basel. The study was approved by the Ethics Committee Beider Basel. Details of the SAKK 38/07 study are reported elsewhere [28].

### In situ biomarker analysis

Immunohistochemical (bcl2, bcl6, c-myc, CD5, CD10, CD95, CD168, cyclin E, FOXP1, GCET, LMO2, MUM1p, pSTAT3) and in situ hybridization analyses [*BCL2* break apart probe (BAP), *C-MYC* BAP and *C-MYC/IGH* double-fusion probe (DFP), and Epstein–Barr virus probe (EBER)] were performed and correlated with clinico-pathological parameters and clinical endpoints. Cell of origin (COO) was determined according to the Tally algorithm [29]. Additionally, selected cases were stained for CD23, CD30, cyclin D1, D2, D3, Ki-67, p27, p63, and SOX11 for specification of diagnosis. Reagent sources, pretreatment and incubation conditions, and cutoff scores are listed in Table 1. Immunohistochemical markers were assessed by microscopic counting of positive cells/tumor cells and were recorded in 5 % increments in the primary statistical table. All cases were scored after training by at least two observers (either AT, SM, or SD), and only markers for which Cronbach’s alpha analysis suggested good agreement between observers (alpha >0.75) were considered for prognostic evaluation. Relevant cutoff scores were either taken from the literature [29, 30] or calculated applying receiver operating characteristic (ROC) analysis [12]. Discrepancies in the results for evaluated markers, which were almost exclusively due to differential assessment of weak staining signals, were discussed at a double-headed microscope and the concordant result was considered. Fluorescence in situ hybridization (FISH) was performed exactly as described elsewhere [31]. All cases were FISH-scored twice (NL and AT) with an excellent agreement (alpha = 1) between both observers.

**Table 1:** Applied biomarker panel

Marker	Source/clone	Pretreatment	Dilution	Incubation	Other	Cutoff (AUROC or reference)
bcl2	Ventana/Roche 790-4604	CC1 16′	RTU	12′		70 % [34, 46]
bcl6	Ventana/Roche 760-4241	CC1 32′	RTU	28′		30 % [30]
c-myc	Ventana/Roche 790-4628	CC1 92′	RTU	16′, 37 °C		40 % [34, 46]
CD5	Ventana/Roche 790-4451	CC1 24′	RTU	12′		20 % (0.542)
CD10	Ventana/Roche 790-4506	CC1 24′	RTU	16′		20 % [29]
CD95	Leica NCL-FAS-310	PC 120 °C, 3′, citrate buffer pH 6	1:400	60′, 20 °C		1 % (0.613)
CD168	Leica NCL-CD168	CC1 extended 92′	1:200	32′	Biotin blocker	10 % (0.536)
					Amplification	
Cyclin E	Thermo MS-1060-S	MW 98 °C, 30′, citrate buffer pH 6	1:20	Overnight, 4 °C		12 % (0.669)
FOXP1	Ventana/Roche 760-4611	CC1 16′	RTU	12′		50 % [45]
GCET	Abcam Ab68889	CC1 32′	1:25	20′		60 % [29]
LMO2	Ventana/Roche 790-4368	CC1 32′	RTU	16′		30 % [29]
MUM1p	Ventana/Roche 760-4529	CC1 24′	RTU	16′		70 % [29]
pSTAT3	Cell Signaling 9145	MW 98 °C, 30′, TEC buffer pH 8	1:50	Overnight, 4 °C	Biotin blocker	17 % (0.602)
*BCL2* BAP	Abbott/Vysis 07 J75-001	Exactly as described [31]				>3 % [31]
*C-MYC* BAP	Abbott/Vysis 05 J91-001					>4 % [31]
*MYC/IGH* DFP	Abbott/Vysis 05 J75-001					>6.5 % [31]
EBER	Ventana/Roche 760-1209	According to the manufacturer’s protocol				10 %

For diagnostic purposes and to “subtract” CD3-positive T cells in CD5-positive DLBCL, CD3 and CD20 stainings were also performed, but these were not considered biomarkers sensu stricto

### Statistics

All statistical analyses were performed using the Statistical Package of Social Sciences (IBM SPSS version 19.0, Chicago, IL, USA) for Windows and reported applying the REMARK guidelines [32]. The inter-observer agreement was assessed using the Cronbach’s alpha reliability analysis; an alpha value of >0.75 indicates very good agreement. The Spearman rank correlation was used to analyze relationships between biomarkers and clinical and laboratory parameters; only correlations with a rho ≥ ±0.300 were considered. The Mann–Whitney *U* and Kruskal–Wallis tests were applied, where appropriate, to identify quantitative differences between groups. The prognostic performance of variables and determination of optimal cutoff values (except those extracted from the most recent literature) was assessed by ROC curve plotting sensitivity versus 1-specificity with special consideration of the respective area under the ROC (AUROC). The optimal cutoff point was calculated using Youden’s index (*Y*), denoting *Y* = sensitivity + specificity − 1, since this method can be applied to find the optimal unbiased cutoff value with the highest sensitivity and specificity [12]. OS was measured from registration to death or last follow-up, PFS from registration to relapse, death of any cause, or to last follow-up, and EFS from registration to relapse or death of any cause, initiation of any non-protocol anticancer treatment because of lymphoma symptoms or need of concomitant radiotherapy or to last follow-up. The probabilities of survival were determined using the Kaplan–Meier method, and differences were compared using the log-rank test. All biomarkers of prognostic significance in univariable models underwent multivariable analysis using the Cox proportional hazards model in a two-step manner since only that response criterion (either according to international criteria or PET or combined PET/CT response) with the highest relevance in an independent first step Cox model, run without biomarkers, was considered and compared to the biomarkers in the second step. All *p* values were two-sided and considered statistically significant if <0.05. No adjustment for multiple testing was applied for secondary analyses because they were considered hypothesis generating and exploratory.

## Results

### Patients, case review, and clinico-pathologic characteristics

Nineteen patients refused a participation in the translational research part of the project. In 11 cases, no material for translational research was present. Thus, 126 cases were further studied: DLBCL diagnosis could not be confirmed in three of these cases by conventional morphology and additional immunohistochemical evaluation (the final diagnosis of marginal zone lymphoma was established in two cases and one turned to be a blastoid mantle cell lymphoma). Thus, the analysis was finally performed on 123 cases. Patient characteristics are given in Table 2. Survival data were complete for 116 patients.

**Table 2:** Basic patient characteristics

Age, median (range)		58 (18–81)
Gender, *N* (%)	F	68 (55)
	M	55 (45)
Stage, *N* (%)	I	12 (10)
	II	41 (34)
	III	30 (24)
	IV	39 (32)
	Missing	1
IPI, *N* (%)	0	23 (19)
	1	36 (29)
	2	27 (22)
	3	20 (16)
	4	13 (11)
	5	4 (3)
Treatment response according to international criteria, *N* (%)	CR	102 (83)
	PR	18 (15)
	SD	2 (2)
	PD	0 (0)
	Missing	1
Combined metabolic and morphologic responses, *N* (%)	Complete metabolic and morphologic response	68 (59)
	Complete metabolic response with residual mass	24 (21)
	Partial metabolic response with residual mass	23 (20)
	Missing	8
Collecting institutions, *N* (%)	University hospitals	33 (27)
	Other hospitals	90 (73)

Eighty-nine lymphomas were primary nodal or of lymphoid tissue (including the mediastinum, the spleen, and Waldeyer’s ring), while 34 were extranodal (most commonly soft tissue, gastrointestinal tract, and bones). Based on integrative analysis, 100 cases were shown to be centroblastic DLBCL, five were immunoblastic DLBCL, three were anaplastic DLBCL, six were unclassifiable, six were primary mediastinal large B cell lymphomas (PMBL; thereof, two were nodal DLBCL with morphologic and phenotypic features of PMBL), two were T cell- and histiocyte-rich B cell lymphomas (THRBCL), and one was a lymphomatoid granulomatosis (LG) grade 3.

The study material consisted of 66 (54 %) lymphadenectomy specimens that were studied on tissue micro-arrays (TMA) and 57 (46 %) cases with only small core needle biopsy material available, which were considered non-arrayable and were studied on conventional serial sections. Arrayable cases were brought into a TMA format applying the 1-mm core needle as described [33].

### In situ biomarkers

**Fig. 1:**
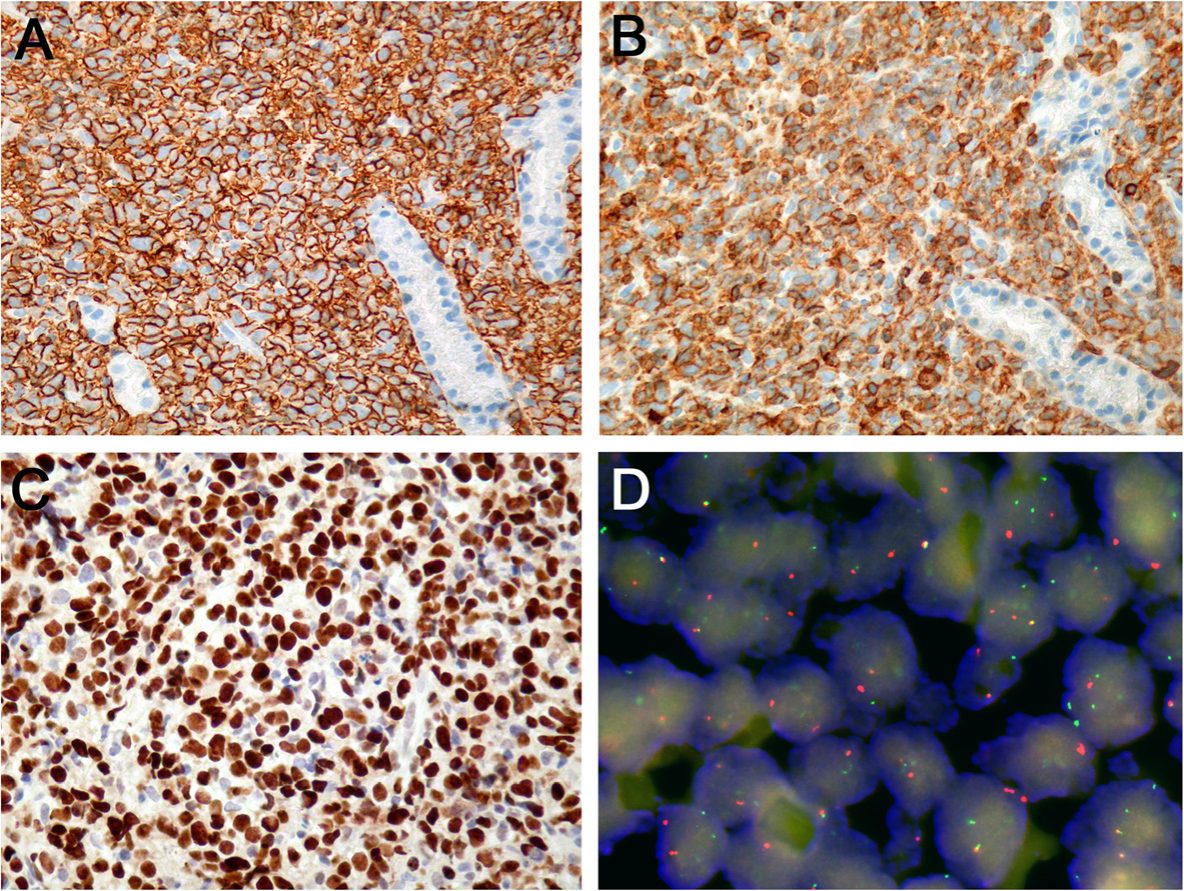
Microphotographs of selected cases. Co-expression of CD20 (**a**) and CD5 (**b**) in an extranodal (intestinal) CD5-positive diffuse large B cell lymphoma. Microphotographs have been taken from consecutive sections; note deeper sections in **b** of the same glandular structures from **a**. Original magnification × 320. **c** Expression of FOXP1 in a positive case. Original magnification × 400. **d** Split *red* and *green signals* corresponding to a *BCL2* gene rearrangement (translocation). Fused *yellow signals* corresponding to the non-translocated allele. Original magnification × 800

Immunohistochemistry was evaluable in all cases, while FISH was successful in 82 (67 %) instances (Table 3, Fig. 1a–d); importantly, cases in which FISH failed were evenly distributed among arrayable lymphadenectomy specimens and small core needle biopsy specimens but were more commonly observed in tissues from certain primary pathology institutions. Taking into consideration the Tally algorithm, 81 cases (66 %) were classified as non-GCB DLBCL, while 42 cases (34 %) were GCB DLBCL; after excluding the PMBL, THRBCL, and LG, there were 39 GCB and 75 non-GCB cases. *BCL2* gene breaks were observed in 7/82 cases (9 %); 6 of the 7 (86 %) rearranged cases were of the GCB type. Two cases (all of the non-GCB type) showed *BCL2* amplifications. *C-MYC* breaks were observed in 6/82 cases (8 %); 4 were of the GCB type. Of the *C-MYC* rearranged cases, only 2 displayed *C-MYC/IGH* fusions, detectable by both DFP and BAP and corresponding to *t*(8;14), while *C-MYC* rearrangements were detectable only by BAP in the other 4 cases and were thus assumed to have occurred with alternative non-*IGH C-MYC* rearrangement partners. “Genetic double-hit” cases with *BCL2* and *C-MYC* rearrangements were not observed.

**Table 3:** Immunohistochemical staining results

	COO	bcl2	c-myc	CD5	CD95	CD168	Cyclin E	FOXP1	pSTAT3	EBER
Evaluable cases	123	123	123	123	123	123	123	123	123	123
Mean % of stained cells ± SD	na	34 ± 38	32 ± 26	2 ± 14	32 ± 42	4 ± 9	8 ± 13	31 ± 38	19 ± 27	na
*N* (%) above cutoff	na	34 (28)	44 (36)	4 (3)	60 (48)	38 (31)	30 (24)	44 (36)	44 (36)	2 (1.5)
Mean % of stained cells ± SD in positive cases	na	94 ± 10	61 ± 20	72 ± 27	65 ± 38	13 ± 13	26 ± 13	79 ± 17	49 ± 26	33 ± 25
Germinal center B cell (GCB) like, *N* (%)	42 (34)	11 (32)^a^	16 (36)^a^	1 (25)^a^	22 (37)^a^	18 (47)^a^	11 (37)^a^	8 (18)^a^	17 (39)^a^	1 (50)^a^

Except for FOXP1, which was of prognostic significance as an isolated marker, all other relevant proteins for cell of origin (COO) classification according to the Tally algorithm are summarized within the COO column

*na* not applicable

^a^GCB out of the positive cases

The presence of *BCL2* breaks correlated with expression of bcl2 (rho = 0.355, *p* = 0.001), CD10 (rho = 0.388, *p* < 0.005), and GCB (rho = 0.302, *p* = 0.006). As expected, expression of GCET, bcl6, CD10, and LMO2 correlated with each other (GCB COO). pSTAT3 correlated with MUM1p, FOXP1, bcl6, and CD168 (rho = 0.301–0.473, *p* = 0.01–0.0001). FOXP1 correlated with bcl2, MUM1p, and c-myc (rho = 0.429, 0.438, and 0.319, respectively, *p* < 0.001). Expression of CD5 did not correlate with any of the examined single variables but showed a weak correlation with the so-called phenotypic bcl2/c-myc double hits (rho = 0.24, *p* = 0.02). Phenotypic bcl2/c-myc double hits [34] correlated with expression of FOXP1 (rho = 0.379, *p* = 0.0002) and *BCL2* rearrangements (rho = 0.319, *p* = 0.005).

### Outcome analysis

The primary study endpoint, i.e., EFS at 2 years, correlated with failure to achieve response according to international criteria and failure to achieve complete combined metabolic and morphologic response or metabolic response (rho values for all >0.470, *p* values for all <1e − 5). The median follow-up period was 53 months (95 % CI 45–51). There were 48 events, including 16 lethal events and 12 relapses 3 months after achievement of CR, of which 6 occurred >12 months after initial diagnosis. The 16 lethal events encompassed 9 deceases with/of disease and 7 deaths unrelated to cancer. Mean OS was 68 months (95 % CI 64–71), mean PFS was 59 months (95 % CI 53–65), and mean EFS was 46 months (95 % CI 40–52); median OS, PFS, and EFS for the whole collective were not reached.

**Fig. 2:**
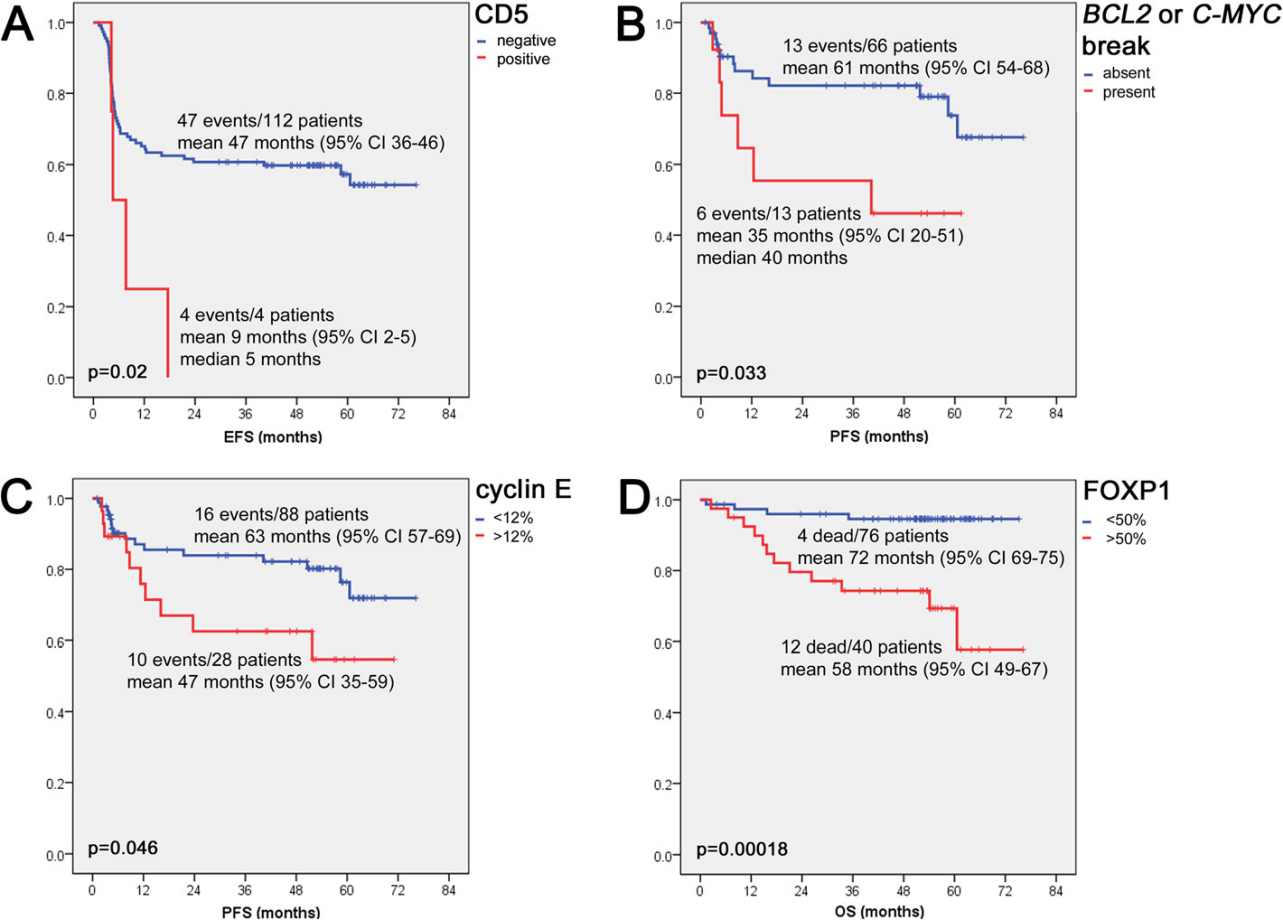
Survival curves. Event-free (EFS) (**a**), progression-free (PFS) (**b**, **c**), and overall survival (OS) (**d**) with respect to biomarker expression

All biomarkers were assessed for their prognostic importance after rational dichotomization (cutoffs listed in Table 1). Factors able to predict worse EFS in univariate Kaplan–Meier models were failure to achieve response according to international criteria, failure to achieve complete combined metabolic and morphologic response or metabolic response (*p* values for all <0.005), expression of CD5 (*p* = 0.02; Fig. 2a), and higher stage (*p* = 0.021). Factors predicting inferior PFS were failure to achieve response according to international criteria, failure to achieve complete combined metabolic and morphologic (but not only metabolic) response (*p* < 0.005), higher IPI (*p* = 0.006), higher stage (*p* = 0.005), presence of either *C-MYC* or *BCL2* gene rearrangements (*p* = 0.033; Fig. 2b), and expression of cyclin E in >12 % of tumor cells (*p* = 0.046; Fig. 2c). Finally, factors predicting inferior OS were failure to achieve response according to international criteria, failure to achieve complete combined metabolic and morphologic (but not only metabolic) response (*p* values for all <0.005), expression of FOXP1 in >50 % of tumor cells (*p* < 0.005; Fig. 2d), expression of cyclin E in >12 % of tumor cells (*p* = 0.005), expression of CD5 (*p* = 0.007), expression of bcl2 in >70 % of tumor cells (*p* = 0.016), expression of CD95 in any tumor cell (*p* = 0.018), and expression of pSTAT3 in >17 % of tumor cells (*p* = 0.025). All other clinico-pathological and phenotypic variables were not of prognostic significance respecting EFS, PFS, and OS. The multivariable analyses’ results for EFS, PFS, and OS are shown in Table 4. Subgroup analysis limited to the DLBCL, not otherwise specified (NOS) cohort (omitting PMBL, THRBCL, and LG because of their more specific biology) revealed that expression of CD5 (*p* = 0.044) retained its independent prognostic significance with respect to EFS (more sensitive for early events) and expression of FOXP1 (*p* = 0.004) with respect to OS (later events), while all other biomarkers failed to add prognostic information. In the case of CD5 because of the only weak correlation of CD5 with phenotypic bcl2/c-myc double hits, the limited number of CD5-positive cases, and the lacking prognostic significance of phenotypic bcl2/c-myc double hits in that series, multivariable analysis was not adjusted for phenotypic bcl2/c-myc double hits. Adjustment for phenotypic bcl2/c-myc double-hit scores in the case of FOXP1 showed that it retained its prognostic significance in those DLBCL, NOS cases scored 0 and 1 (and outperformed failure to achieve combined metabolic and morphologic remission in cases scored 0), but neither expression of FOXP1 nor failure to achieve complete combined metabolic and morphologic remission were of prognostic significance with respect to OS in phenotypic bcl2/c-myc double-hit score 2 DLBCL, NOS cases (data not shown in detail).

**Table 4:** Multivariable analysis

Survival	Parameter	Hazard ratio	95 % CI	*p* value
Event-free	Lack of complete combined metabolic and morphologic remission	2.54	1.84–3.51	<0.005
	Expression of CD5	2.99	1.02–9.13	0.047
Progression-free	Lack of complete remission according to international criteria	16.39	4.57–58.82	<0.005
Overall	Expression of FOXP1	5.61	1.34–23.4	0.018
	Lack of complete combined metabolic and morphologic remission	1.93	1.04–3.58	0.038

Only significant results are shown

**Fig. 3:**
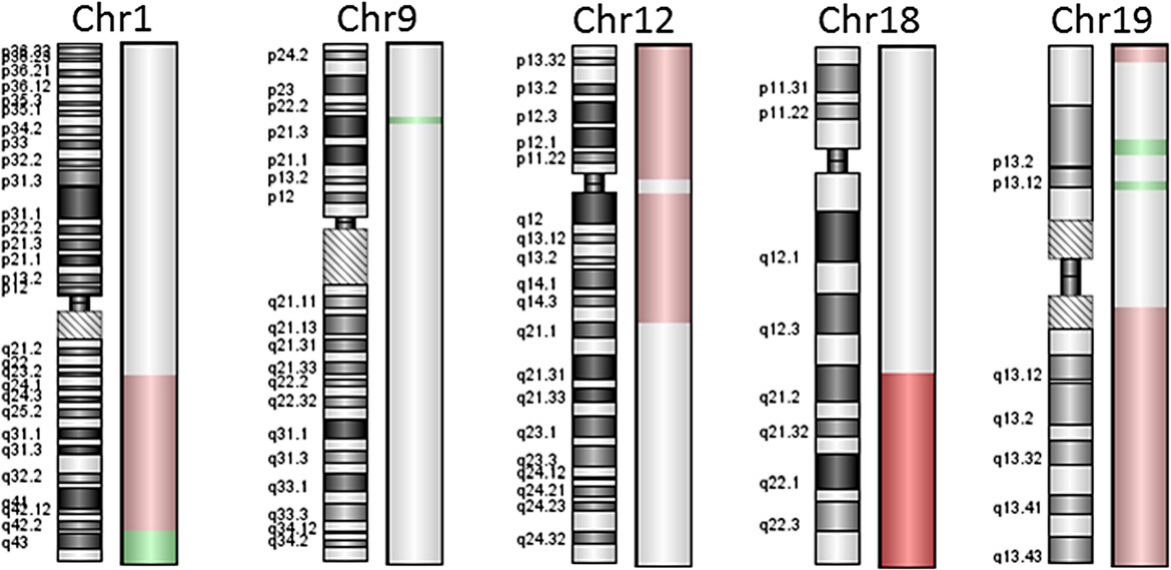
Copy number aberrations in a CD5-positive case. Only aberrant chromosomes are shown. *Green* represents losses, and *red* represents gains of DNA. *Darker red* in the long arm of chromosome 18 indicates high-level amplification of this region. Note the small deletion at 9p21.3 (*INK4A* locus), known to be associated with chemoresistance

Since CD5 expression appeared to be of significant relevance, we thoroughly revised the four CD5-positive cases and evaluated multiple immunohistochemical markers to exclude blastoid mantle cell lymphomas (shown above). The four CD5-positive DLBCL were negative for cyclin D1 and SOX11 and expressed p27. These cases stained positively for CD5 in 50 to 100 % of tumor cells did not show an intravascular component and were negative for EBER; three were classified as non-GCB, while one was GCB; and three showed centroblastic morphology, while one was classified as centroblastic with increased immunoblasts. None of these four CD5-positive cases showed presence of either *C-MYC* or *BCL2* gene rearrangements; however, two patients fulfilled phenotypic criteria for double-hit lymphoma, expressing bcl2 or c-myc above the respective cutoff scores. Two patients were male; two suffered from nodal lymphomas; two were Ann Arbor stage II, while the other two were stage I and III, respectively; and two patients had an IPI of 1 and two an IPI of 2. The mean age of the CD5-positive patients was 64 ± 13 years, while that of the CD5-negative was 58 ± 13 (difference not of statistical significance). Two of the four patients failed to achieve remission (one of these two patients died of/with lymphoma) and in the other two DLBCL relapsed after 8 and 38 months, respectively. Finally, DNA of the four CD5-positive cases was extracted and subjected to array comparative genomic hybridization (aCGH) analysis (Fig. 3) exactly as described elsewhere [35]. The analysis was successful in two cases and showed recurrent gains of 19q and losses of 1q43 [36], thus further corroborating the diagnosis of DLBCL. One of the cases showed specific loss of 9p21 (*INK4A* locus, also known as p16) known to be associated with DLBCL resistance to R-CHOP [37].

## Discussion

Within this prospective study, we identified potential biomarkers (expression of CD5 for EFS and expression of FOXP1 for OS) that were able to predict the course of DLBCL at diagnosis, independent of stage and IPI. As expected ([38] and literature therein), dynamic parameters, such as response to therapy and especially failure to achieve complete remission, which are not obtainable at diagnosis, seem to be the most reliable outcome indicators in DLBCL, yet expression of CD5 and FOXP1 added information independent of these disease dynamic parameters.

Concerning the central aim of our study, i.e., to detect in situ biomarkers that reliably help predicting the outcome of DLBCL in a prospective, homogeneously treated collective of patients, our phenotypic and genotypic analyses show that carefully selected indicators such as CD5 might identify small yet prognostically relevant subgroups with adverse outcomes under R-CHOP. CD5 as biomarker has a special sensitivity towards early adverse events, which might not be the case for some of the currently propagated biomarkers of prognostic relevance such as c-myc expression/*C-MYC* gene status. Furthermore, our data reappraise the prognostic role of FOXP1 with respect to OS. Several other previously studied biomarkers with suspected prognostic potential like COO, expression of bcl2, or phenotypic double-hit score appeared to be less potent in the studied collective. This might in part be due to the small size of our study, in part to genuine properties of these markers, and in part to the fact that some of these markers, while being applicable to CHOP-treated DLBCL patients, are not applicable to cases treated with R-CHOP [39]. Considering our study size, there are obvious and inevitable limitations. Yet, because of the other characteristics of our collective (123 uniformly treated patients with a median follow-up period of 53 months and altogether 51 adverse events), our data solidifies understanding of the prognostic importance of in situ biomarkers in DLBLC and the 2-year EFS analysis delivers important results. Respecting the genuine properties of some markers, especially those used as surrogates to determine COO, our results as well as observations of others [14] seriously challenge their reliability to identify prognostically and/or biologically meaningful groups among DLBCL.

Our observed prognostic role of CD5 and FOXP1 and possible prognostic role of bcl2 as well as structural genetic aberrations of (either) *BCL2* or *C-MYC* are supported by other reports ([31, 40–46] and literature therein). While a considerable number of recent papers focused on the role of bcl2 and c-myc in DLBCL [34, 46, 47], it seems that CD5 merits special attention for several reasons: (a) it can be very easily detected in DLBCL by standard application of CD5 (instead of CD3) immunohistochemistry in the primary diagnostic panel with subsequent application of CD3 in CD5-positive cases (to subtract the “true” T cells), as well as CD23, cyclin D1, and SOX11 (to exclude transformed small lymphocytic B cell lymphomas and blastoid mantle cell lymphomas); (b) the respective cases express CD5 in a high proportion of tumor cells (>50–100 %) with a moderate to strong staining intensity, and thus, its evaluation is unequivocal without the need for subjective and error-prone cutoff scores; and (c) because there is an increasing body of literature suggesting that CD5-positive DLBCL might represent a distinct biologic entity, being more prone to intravascular spread and extranodal location (particularly CNS), affecting individuals from the Far East and displaying a more aggressive behavior probably requiring alternative treatment approaches [40]. CD5-positive DLBCL are typically ABC [42, 48], show recurrent gains of 16p and losses of 1p and of 9q21 [36, 49], the latter being involved in chemoresistance [37], and display downregulation of extracellular matrix-related genes and upregulation of neurological function-related genes [48]. Addition of rituximab to CHOP improved the survival of CD5-positive DLBCL patients [50]; however, similarly to our results, the outcome of these patients is still significantly poorer compared to CD5-negative DLBCL patients [51], and the rate of CNS involvement seems not to be lowered by rituximab [52]. A recent very large retrospective report on 879 R-CHOP-treated DLBCL cases convincingly showed CD5 to be an IPI (and bcl2 and pSTAT3)-independent prognosticator in DLBCL as well [53] and pointed out distinct clinico-pathological peculiarities of such patients such as increased age, bone marrow spread, poor performance status, and B symptoms. Considering the possible direct biological effect of CD5 on B cells, namely its role as a negative regulator of B cell signaling, its influence on the ERK, PI3K, and calcineurin pathways as well as survival stimulation through autocrine IL10-related loops and the predominant expression of integrin beta-1 on the tumor cells, CD5 seems to be of probable functional and therapeutic importance for targeted approaches [40, 54–56]. In addition, CD5-positive cases seem to overexpress bcl2, CARD11, CCND2, and FOXP1 at the protein and mRNA level and to be more rich in c-Rel, p65, and pSTAT3 [53], all known to identify DLBCL patients at risk; this study [53] also confirmed [48] downregulation of cellular adhesion genes in such instances. Taken together, previous data and our observations might justify a separation of CD5-positive DLBCL out of the group of DLBCL, NOS, as a distinct clinico-pathological entity in need of R-CHOP treatment alternatives and, probably, CNS prophylaxis.

The prognostic role of FOXP1 in DLBCL was well established in the “pre-rituximab” era ([45] and references therein), while less attention has been paid to it in R-CHOP-treated cases. Importantly, prognostically relevant COO algorithms pay special attention towards expression of FOXP1 to classify non-GCB-like DLBCL and >90 % concordance with GEP was only achievable by consideration of FOXP1 in these algorithms (e.g., [29, 44]). In line with these results, the recent report on the very poor prognosis of DLBCL reciprocally expressing the endocytic protein Huntingtin-interacting protein 1-related (HIP1R) and FOXP1 (the latter being a direct repressor of the *HIP1R* gene), i.e., FOXP1(hi)/HIP1R(lo) patients [57], and our prospective study findings suggest a more substantial relevance of FOXP1 in DLBCL. Importantly, FOXP1 belongs to the most reproducibly assessable markers in DLBCL as shown in an international inter- and intra-institutional and inter- and intra-observer study [58], further calling for its regular evaluation.

Unexpectedly, a significant (33 % for FISH and 50 % for aCGH) dropout of cases for genotypic studies was noted. Detailed analysis of these cases revealed that pre-analytic conditions like inappropriate application of un-buffered formalin, fixation duration, surrounding temperature, and exact dehydration procedures were probably more relevant for lack of analytic success than the exact amount of examined tissue. Indeed, these failures were evenly distributed between core needle biopsies and lymphadenectomy specimens but were more commonly observed among tissues from a few centers. As expected, diagnostic tissue obtained by core needle biopsy procedures (usually 14–18G needles) was not arrayable and was rapidly exhausted for purposes of the study, precluding further analyses. Since cohorts of prospective clinical trials are characterized by meticulous documentation and uniform treatment of patients (the latter, if not uniform, can more substantially affect disease prognosis than many biomarkers), biomarker analyses should desirably be performed on cases collected within such studies. Therefore, the amount and the pre-analytical handling of tissue required for study inclusion must be considered also under the aspect of biomarker analyses. This particularly implies that physicians obtaining and handling the respective biopsies as well as the pathology laboratories must take responsibility for error-free and safe pre-analytic conduits, guaranteeing optimal tissue fixation and dehydration, which are indispensable for an accurate morphologic, phenotypic, and genetic analysis. For practical purposes, the protocol for probe handling from the laboratory, which provided probes with least dropout on molecular testing, is given in Additional file 1: Table S1.

## Conclusions

In summary, distinct biomarkers like CD5 and FOXP1 are able to prognosticate DLBCL course at diagnosis, independent of stage and IPI and independent of initial therapy response. For the design of prospective DLBCL studies, issues like review of the slides by a central pathology, pre-analytic factors such as time to and time of fixation, choice of fixative, and dehydration as well as handling of biological entities and sub-entities in the spectrum of aggressive large B cell lymphomas should be properly discussed and promptly addressed.

## Additional file

Additional file 1: Table S1. Summary of pre-analytics in the lab, submitting probes with least number of molecular testing dropouts.

## Abbreviations

ABC: Activated B cell
aCGH: Array comparative genomic hybridization
AUROC: Area under the ROC curve
BAP: Break-apart probe
CNS: Central nervous system
COO: Cell of origin
DFP: Double-fusion probe
DLBCL: Diffuse large B cell lymphoma
ECOG: Eastern Cooperative Oncology Group
EFS: Event-free survival
FISH: Fluorescence in situ hybridization
GCB: Germinal center B cell
G-CSF: Granulocyte colony-stimulating factor
GEP: Gene expression profiling
HIV: Human immunodeficiency virus
IPI: International Prognostic Index
LG: Lymphomatoid granulomatosis
MRI: Magnetic resonance imaging
NOS: Not otherwise specified
OS: Overall survival
PET: Positron emission tomography
PSF: Progression-free survival
PMBL: Primary mediastinal B cell lymphoma
R-CHOP: Rituximab, cyclophosphamide, hydroxydaunorubicin, vincristine, prednisone
R-IPI: Revised International Prognostic Index
ROC: Receiver operating characteristic
THRBCL: T cell- and histiocyte-rich B cell lymphomas
TMA: Tissue microarray
