# Supplementary material for: Multiparameter analysis of homogeneously R-CHOP-treated diffuse large B cell lymphomas identifies CD5 and FOXP1 as relevant prognostic biomarkers: report of the prospective SAKK 38/07 study
Source: J Hematol Oncol. 2015 Jun 14;8:70. doi: 10.1186/s13045-015-0168-7 (PMC4472251; doi:10.1186/s13045-015-0168-7)
Supplement: Additional file 1: Table S1. — Summary of pre-analytics in the lab, submitting probes with least number of molecular testing dropouts. [file 13045_2015_168_MOESM1_ESM.doc]

Additional file 1

| **Table S1.** Summary of pre-analytics in the lab, submitting probes with least number of molecular testing drop-outs | | | |
| --- | --- | --- | --- |
| Procedure | Solution | Duration | Commercial product/machine |
| Fixation, core needle biopsies | 4% buffered formaldehyde solution | 24h | Formafix |
| Fixation, ectomies | 4% buffered formaldehyde solution  tissues are fixed after having been sliced into 3mm thick and 1.5x1.5cm large pieces | 24h | Formafix |
| Dehydration, core needle biopsies | Ethanol 70%  Ethanol 80%  Ethanol 96%  Ethanol 100%  Xylol  Xylol | 2 x 1h at 37°C  1h at 37°C  2 x 1h at 37°C (vacuum pump)  2 x 1h at 37°C (vacuum pump)  2 x 0.5h at 37°C (vacuum pump)  1h at37°C (vacuum pump) | Automatic device |
| Paraffin infiltration, core needle biopsies |  | 3 x 1h at 60°C (vacuum pump) |
| Dehydration, ectomies | Additional formalin fixation  Ethanol 70%  Ethanol 80%  Ethanol 96%  Ethanol 100%  Ethanol 100%  Xylol  Xylol | 3/4h at 45°C  2 x 0.5h at 45°C  1 x 0.5h at 45°C  0.5h at 45°C  1h at 45°C  1.5h at 45°C  2 x 3/4h at 45°C  1.5h at 45°C | Automatic device |
| Paraffin infiltration, ectomies |  | 2 x 1h at 62°C  1.3h at 62°C |
